# Difficult decisions: Evaluating individual and couple-level fertility intentions and HIV acquisition among HIV serodiscordant couples in Zambia

**DOI:** 10.1371/journal.pone.0189869

**Published:** 2018-01-24

**Authors:** Dvora Leah Joseph Davey, Kristin M. Wall, William Kilembe, Naw Htee Khu, Ilene Brill, Bellington Vwalika, Elwyn Chomba, Joseph Mulenga, Amanda Tichacek, Pamina M. Gorbach, Susan Allen

**Affiliations:** 1 Rwanda Zambia HIV Research Group, Department of Pathology & Laboratory Medicine, School of Medicine and Hubert Department of Global Health, Rollins School of Public Health, Emory University, Atlanta, GA, United States of America; 2 Department of Epidemiology, Fielding School of Public Health, UCLA, Los Angeles, California, United States of America; 3 Department of Biostatistics and Epidemiology, School of Public Health and Family Medicine, University of Cape Town, Cape Town, South Africa; 4 Department of Epidemiology, Rollins School of Public Health, Laney Graduate School, Emory University, Atlanta, Georgia, United States of America; 5 Department of Epidemiology, Ryals School of Public Health, University of Alabama at Birmingham, Birmingham, Alabama, United States of America; 6 Department of Gynecology and Obstetrics, School of Medicine, University of Zambia, Lusaka, Zambia; 7 Department of Pediatrics, School of Medicine, University of Zambia, Lusaka, Zambia; Tulane University School of Public Health and Tropical Medicine, UNITED STATES

## Abstract

**Introduction:**

Attempts to conceive and pregnancy may increase HIV transmission to sex partners and infants. Our study evaluated the association between fertility intentions and HIV acquisition among Zambian HIV-serodiscordant couples.

**Methods:**

We collected demographic, behavioral, clinical exposures, and data on fertility intentions in a cohort of HIV-serodiscordant couples in Lusaka, Zambia from 2005 to 2012. We evaluated factors associated with fertility intentions stratified by gender using multivariable logistic regression. Multivariable Cox proportional hazard models were used to evaluate the associations between fertility intentions and HIV acquisition controlling for *a priori* confounders and covariates that substantially (>10%) changed the effect estimates in univariate analyses.

**Results:**

Among 1,029 serodiscordant couples, 311 agreed that they wanted children in the future (30%), 368 agreed they did not want children (36%), and 344 couples disagreed about having children (34%), with men more likely than women to want children. Women wanting child(ren) was associated with increased odds of baseline pregnancy (adjusted odds ratio [aOR] = 4.80 (95% confidence interval [CI] = 2.93, 7.85)), fewer previous pregnancies (aOR = 0.85 per additional pregnancy (95% CI = 0.78, 0.93)), and partner fertility intention (aOR = 2.89 (95% CI = 2.14, 3.91)) adjusting for woman’s age, literacy, years cohabiting and HIV status. Men wanting child(ren) was associated with younger age (aOR = 0.96 per year (95% CI = 0.93, 0.99)), fewer years cohabiting (aOR = 0.95 (95% CI = 0.92, 0.98)), number of previous partners’ pregnancies (aOR = 0.90 (95% CI = 0.82, 0.98)), and partner fertility intention (aOR = 3.00 (95% CI = 2.21, 4.07)) adjusting for partner’s age, literacy, HIV status and partner’s baseline pregnancy. In adjusted survival analyses, HIV-negative women were more likely to seroconvert if they themselves wanted children (aHR = 2.36 (95% CI = 1.41, 3.96)) vs. did not want children, or if their partner wanted children (aHR = 2.34 (95% CI = 1.33, 4.11)) vs. did not want children, or if the couple agreed that they wanted children (aHR = 2.08 (95% CI = 1.01, 4.30)), adjusting for women’s age, women’s literacy, previous pregnancies and time in study. HIV-negative men were more likely to seroconvert if their female partner wanted a child in the next 12-months (aHR = 1.94 (95% CI = 1.02, 3.68)) vs. did not want children, and when both partners wanted children (aHR = 2.02 (CI = 1.09, 3.73)) vs. they did not want children, adjusting for men’s age and literacy, couple income, number of live children, male circumcision status and time in study.

**Conclusion:**

Women had increased risk of HIV acquisition if they and/or their partner wanted a child, while men had increased risk of HIV acquisition when their partner or if both partners agreed that they wanted children. Safer-conception interventions are needed to protect HIV uninfected women and men from HIV acquisition in HIV-serodiscordant couples who want children.

## Introduction

Of the estimated 33-million people living with HIV worldwide, 16-million are women [1]. Since the introduction of antiretroviral therapy (ART), pregnancy rates and the intention to conceive have increased among women and men living with HIV, due in part to improved quality of life, increased life expectancy, and reduced rates of mother to child transmission [2–4]. Attempts to conceive and pregnancy may increase HIV transmission to sex partners in HIV serodiscordant couples (in which one partner is HIV-infected (HIV+) and the other HIV uninfected (HIV-) [2–4].

Previous research on the determinants of fertility intentions in HIV+ women, and serodiscordant couples has yielded mixed results [5–9]. Men and women of younger age and those without children are more likely to intention (more) children [8, 9]. However, intention to have children may also be influenced by gender, access and adherence to ART, knowledge of prevention of mother to child transmission of HIV programs (PMTCT), stigma, and social support as well as family, culture and gender norms [8–10]. A recent study in Zambia by Cook et al. found that intention to have children was often shared by couple members, and among serodiscordant couples or concordant couples the strongest predictor of a partner’s intention for children was having a partner who wanted children [10]. Addressing the varied needs of women of reproductive age who are living with HIV, or are at risk of acquiring HIV, as in the case of serodiscordant couples, is a complicated task that requires improved access to, utilization and quality of comprehensive healthcare.

Safer conception interventions include: timed condomless intercourse (to coincide with ovulation period) [11], intravaginal or intrauterine insemination for couples in which the female is HIV-infected, ART for HIV-infected partners [12–14], and pre-exposure prophylaxis (PrEP) for HIV-negative partners [14], and sperm washing followed by insemination for couples in which the male is HIV-infected [13–18]. While many of these strategies are widely available (except for sperm washing and PrEP), policy makers have not identified safer conception as an important clinical issue for investment. As a result, safer conception focused programming and counseling is not available where the clear majority of serodiscordant couples reside. Further, preventing unintended pregnancies among HIV+ women and serodiscordant couples who don’t want children, through family planning and modern contraception, reduces pregnancy-related morbidity and mortality, decreases the number of pediatric HIV infections, and has also proven to be a cost-effective way to prevent mother-to-child HIV transmission [7, 14].

This study examined the role of fertility intention as a risk factor for HIV acquisition and transmission among serodiscordant couples. We evaluated factors associated with fertility intention among men and women in HIV serodiscordant couples. We then evaluated the effect of the female partner, male partner and both partners wanting more children on HIV acquisition over time.

## Methods

The Heterosexual Transmission of HIV Study conducted by the Rwanda Zambia HIV Research Group (RZHRG) was an open prospective cohort, which enrolled 3,049 adult heterosexual serodiscordant couples recruited from couples’ voluntary counseling and testing (CVCT) centers in Lusaka, Zambia. Data collection began January 1995 and ended December 2012.

### Ethics

This study was approved by the Office for Human Research Protections-registered Institutional Review Boards at Emory University and the University of Zambia (IRB00001131 of IORG0000774). Written informed consent was obtained from all participating individuals in the couple.

### Study participants

Married or cohabiting couples in Lusaka, Zambia, attended CVCT services spontaneously, or after receiving an invitation from a community promoter [19, 20]. CVCT services included a group counseling session followed by rapid HIV testing, and joint post-test couples’ counseling. Eligible HIV serodiscordant couples were then invited to enroll in a longitudinal open cohort follow-up study between 1995 and 2012 [19–21].

Study eligibility included: (1) confirmed HIV-1 serodiscordance (one partner confirmed HIV+ and one partner confirmed HIV-) (2) participants were married or cohabitating for at least three months, and (3) the couple planned on staying in the Lusaka region for the next 12-months. Couples were ineligible if the HIV+ partner was on ART, which became available in government clinics in 2007 with CD-4 cell count eligibility criteria changing over time thereafter. Couples were censored if either partner died, the couple separated, the HIV+ partner initiated ART, or if either partner was lost to follow-up.

### Data collection

At baseline, study participants completed behavioral assessments and medical history questionnaires, and had a full physical examination including pelvic/genital exams, as well as HIV and sexually transmitted infections (STI) testing and treatment. Quarterly follow-up visits included a physical exam, a blood sample for HIV testing (HIV- partner), a vaginal swab wet prep (trichomonas and sperm), and completion of study questionnaires, comprising questions on demographic, psychosocial, behavioral, medical history, and health services data. Couples were asked to complete a coital diary to record number of sex acts with a condom and without. The data was collected for men and women separately through face-to-face interviews by a study counselor or nurse. All data collection instruments were in English and were translated into local languages Nyanja and Bemba.

#### HIV testing

HIV testing of HIV- partners was conducted at three-monthly intervals using a rapid serologic test. To determine time of incident infection, when available, plasma from the last antibody negative sample was tested with p24 ELISA and RNA polymerase chain reaction. The date of HIV infection was defined using available laboratory results as the minimum of: the midpoint between the last negative and first positive antibody date; two-weeks prior to the first antigen positive test date; or two-weeks prior to the first positive viral load positive/rapid HIV test negative test date.

#### Fertility intention exposure measurement

Data on fertility intention was collected at one time point at study enrollment from 2005 to 2012, when the study ended. The first measure was “Do you want to have a child?” If the respondent answered “yes”, then they were asked whether they “want a child in the coming year”, or “yes, but not in the next year”. If the participant (or his partner) were pregnant, the question referred to a future child and did not refer to the current pregnancy.

### Analyses

Analyses included data from men and women separately who reported fertility intentions at baseline. We included 1283 women who reported fertility intentions and 1029 men who reported fertility intentions in our analyses. For couple level analyses the number of couples was 1029 (removing 254 couples in which men did not report fertility intentions). For predictors of fertility intentions, we included couples who did not have missing fertility intention or other covariates, including baseline and incident pregnancy (198 couples were missing one or more indicator in the female model and 196 couples were missing one or more indicator in the male model). In the Cox proportional hazard model we stratified by HIV serostatus and included 545 M+F- couples and 575 M-F+ couples and the final model (n = 1029 couples) in which both partners reported fertility intentions.

Descriptive statistics express baseline demographic and behavioral data by fertility intention. We developed binary outcomes combining (1) Yes, in the coming year, and (2) Yes, but not in the coming year vs. no with counts and percentages (for categorical variables), or means and standard deviations (for continuous variables).

We first analyzed factors associated with wanting a child by serostatus and gender. We then specified logistic regression models for factors associated with wanting a child by gender. Finally, we analyzed seroconversion stratified by gender of seroconverter as a result of the woman, man, or couple wanting a child vs. those not wanting a child. We also compared concordant intention in wanting a child in the couple, vs. disagreement in the couple (man wants more children and woman does not or woman wants more children and man does not).

We developed Cox proportional hazard regression models to calculate time to seroconversion by gender of seroconverter and test the association with male or female (or both) fertility intentions and to test if incident pregnancy was associated with HIV acquisition in females. Fertility intentions were collected at baseline, before HIV acquisition in the model. The proportional hazards assumption was tested for time-independent covariates using Kaplan-Meier curve. Since prior research shows that couples counseling and testing is associated with reduction in HIV incidence [22], all survival models included a variable accounting for the first follow-up interval vs. subsequent follow-up intervals. The highest seroconversion rates were in the first follow-up interval, reflecting incident infections acquired prior to CVCT and in the seronegative window period at the time of first testing. *A priori* confounders (e.g. age, woman’s literacy, number of live children for men, and number of previous pregnancies among women at baseline) and covariates that substantially (>10%) changed the effect estimates in univariate analyses were considered as potential confounders in the multivariable models. STIs were associated with the exposure (fertility intentions) and the outcome (HIV acquisition) in our analysis and prior studies [21], and are an intermediate variable on the causal path between the exposure and outcome. As such we did not control for STIs in our model as it would cause an over adjustment bias, defined as “control for an intermediate variable on a causal path from exposure to outcome.” [23] Effect-measure modification was assessed for gender of the HIV+ partner. Multi-collinearity was assessed by analyzing the variance inflation factor and tolerance to check the degree of collinearity between variables. All analyses were conducted with SAS v9.4 (Cary, NC).

## Results

Our study followed 1,029 couples for an average of 743 days (standard deviation [SD] = 837). Overall, 44% of couples had less than 1 year of follow-up, 22% had between 1 and 2 years of follow up, and 34% had 3 or more years. About half of men wanted to have a child in the next year (56% of HIV- men and 45% of HIV+ men) and 42% of HIV- women and 51% of HIV+ women wanted a child. For couple-level analyses, of 1,029 couples, 42% of M+F- couples agreed that they wanted to have a child in the future, and 26% agreed they do not want a child. Of M-F+ couples, 34% of couples agreed they wanted a child in the future while 31% agreed they did not want a child. Almost 34% disagreed about whether or not to have a child.

We compared the baseline covariates between women (Table 1) and men (Table 2) who did and did not want children, stratified by couple serostatus. HIV- women in discordant couples (M+F-) who wanted children were younger (mean age of 26.4 vs. 31.0, p<0.0001), had lived with their partner fewer years (5.5 vs. 10.0 years, p<0.0001), had fewer pregnancies (2.5 vs. 4.3 mean pregnancies, p<0.0001), and fewer live children (1.4 vs. 2.6 mean live children, p<0.0001) compared with HIV- women in discordant couples (M+F-) who did not want to have more children. During the study follow-up, HIV+ women who wanted a child at baseline reported greater number of condomless sex acts per quarter (2.5 vs. 1.4 mean condomless sex acts and 31% vs. 23% 3-month intervals with reported condomless sex, p<0.0001), more genital inflammation (7% vs. 5%, p<0.0001) and greater incident pregnancies during follow up (12% vs. 5%, p<0.0001) compared with HIV- women in M+F- couples who did not want a child.

**Table 1:** Descriptive analyses of socio-demographic, behavioral, biomedical covariates by fertility intention of Zambian women in serodiscordant couples stratified by serostatus (n = 1283).

		HIV-negative women (M+F- couples)		HIV-positive women (M-F+ couples)	
	Total	Woman does not want a child	Woman wants a child	Woman does not want a child	Woman wants a child
**Total couples**	**1283**	**315 (58%)**	**225 (42%)** **	**367 (49%)**	**376 (51%)**
**Follow-up time (mean days, SD)**	747 (847)	720 (687)	616 (608)**	764 (697)	664 (662)
**Demographics**					
**Woman age (mean, SD)**	28.9 (7.0)	31.0 (7.0)	26.4 (5.8) **	30.8 (6.5)	27.7 (5.9) **
**Age disparity (mean, SD)**	6.9 (4.4)	7.0 (4.4)	7.2 (4.3)	6.4 (5.0)	6.2 (4.3)
**Years cohabiting (mean, SD)**	8.0 (6.6)	10.0 (7.3)	5.5 (4.5) **	7.4 (6.4)	5.1 (4.6) **
**Monthly family income: USD (mean, SD)**	88.6 (112.4)	96.2 (110.8)	92.9 (109.1)	90.6 (119.3)	101.4 (124.7)
**Woman reads Nyanja (vs. illiterate)**	301 (24%)	81 (26%)	36 (16%)*	97 (27%)	87 (23%)
**Man reads Nyanja (vs. illiterate)**	567 (44%)	142 (46%)	92 (41%)	157 (43%)	176 (47%)
**Woman heavy alcohol use last year (vs. none)**	53 (4%)	8 (3%)	7 (3%)	21 (6%)	17 (5%)
**Pregnancy history**					
**Previous pregnancies (mean, SD)**	3.6 (2.4)	4.3 (2.4)	2.5 (1.7) **	3.7 (2.2)	2.7 (1.9) **
**# of live children (mean, SD)**	2.1 (1.8)	2.6 (1.9)	1.4 (1.3) **	1.7 (1.5)	0.9 (1.1) **
**Pregnant at baseline (vs. not pregnant)**	125 (10%)	13 (4%)	45 (20%) **	18 (5%)	49 (13%) **
**Non-barrier contraceptive method at baseline (n = 1277)**					
None/condoms only	762 (60%)	162 (51%)	135 (60%)	202 (55%)	263 (71%) **
Implant	58 (5%)	13 (4%)	11 (5%)	27 (7%)	7 (2%) **
Injectable	239 (19%)	76 (24%)	39 (17%)	75 (21%)	49 (13%) **
Oral contraceptive pills	218 (10%)	64 (20%)	40 (18%)	61 (17%)	53 (14%) **
**Genital symptoms, sexual behaviors and condom use (n = # of intervals in which the variable was measured)**					
**# of times sex with partner in study** **with** **a condom in the last 3-months (mean, SD) (n = 10,415)**	20.3 (21.2)	19.0 (18.4)	20.2 (21.2)	21.9 (22.1)	24.1 (25.3) **
**# of times sex with partner in study** **without** **a condom in the last 3-months (mean, SD) (n = 10,415)**	3.0 (10.3)	1.4 (5.3)	2.5 (8.1) **	2.7 (8.7)	4.8 (15.6) *
**Sex with study partner** **with** **a condom in past 3-months (n = 11,651)**	10,253 (88%)	2905 (88%)	1803 (89%)	2934 (88%)	2611 (87%)
**Sex with study partner** **without** **a condom in past 3-months (n = 11,663)**	3583 (31%)	770 (23%)	619 (31%) **	1097 (33%)	1097 (36%) *
**Genital ulcer in past 3-months (n = 11,425)**	648 / 11,425 (6%)	104/3297 (3%)	59/2044 (3%)	253/3233 (8%)	232/2861 (8%)
**Genital inflammation past 3 months (n = 11,417)**	945/11,417 (8%)	156 (5%)	150 (7%) **	304 (9%)	335 (12%)*
**Sperm ever present on wet prep (n = 11,264)**	336 (3%)	74 (2%)	67 (3%) *	84 (3%)	111 (4%)*
**Outside sex partner (n = 10,770)**	129 (1%)	22 (1%)	33 (2%)*	30 (1%)	44 (2%)*
**Pregnant during follow-up (n = 10,221)**	770 (8%)	162 (5%)	223 (12%) **	168 (6%)	217 (9%) **

**p = <0.0001

*p<0.05

no asterisk = p>0.05 comparing women who want a child vs. those who do not by serostatus

**Table 2:** Descriptive analyses of socio-demographic, behavioral, biomedical covariates by fertility intention of Zambian men in serodiscordant couples stratified by serostatus (n = 1029).

		HIV-positive men (M+F-)		HIV negative men (M-F+)	
	Total	Man does not want a child	Man wants a child	Man does not want a child	Man wants a child
**Total couples**	**1029**	**249 (55%)**	205 (45%)*	**254 (44%)**	**321 (56%)** **
**Follow-up time (mean days, SD)**	745 (842)	775 (701)	747 (662)	810 (708)	812 (752)
**Demographics **					
**Man age (mean, SD)**	35.1 (8.1)	38.1 (7.5)	33.0 (6.7) **	38.0 (8.8)	32.7 (6.6) **
**Age disparity (mean, SD)**	6.8 (4.8)	7.2 (4.4)	7.1 (4.3)	6.9 (5.4)	6.0 (4.2) *
**Years cohabiting (mean, SD)**	6.8 (6.3)	10.4 (7.3)	5.7 (4.9) **	8.1 (7.0)	4.9 (4.1) **
**Monthly family income (USD) (mean, SD)**	90.2 (113.7)	80.0 (81.5)	87.5 (92.3)	90.5 (119.2)	76.5 (101.2)
**Man reads Nyanja (vs. illiterate)**	583 (57%)	117 (47%)	76 (37%)*	119 (47%)	131 (41%)
**Man’s heavy alcohol use last year (vs. none)**	185 (18%)	43 (17%)	38 (19%)	51 (20%)	53 (17%)
**Pregnancy history **					
**# previous pregnancies spouse (mean, SD)**	3.4 (2.3)	4.3 (2.5)	2.7 (1.8) **	3.9 (2.2)	2.7 (1.9) **
**# live children (mean, SD)**	1.7 (1.6)	2.7 (1.8)	1.4 (1.4) **	1.8 (1.6)	0.9 (1.0) **
**Partner pregnant at baseline (vs. not pregnant)**	124 (12%)	22 (9%)	35 (17%) **	26 (10%)	41 (13%)
**Genital symptoms, sexual behaviors and condom use (n = # of intervals in which the variable was measured)**					
**Genital inflammation in past 3-months (n = 9242)**	369/9242 (4%)	127/1919 (7%)	100/1541 (7%)	46/2488 (2%)	96/3294 (3%) *
**Genital ulcer in past 3-months (n = 9245)**	763/9245 (8%)	217/1920 (11%)	211/1543 (14%) *	132 (5%)	203 (6%)
**# of times sex with partner in study** **with** **a condom in the last 3-months (mean, SD) (n = 10,415)**	20.3 (21.2)	18.8 (19.1)	20.4 (21.3) *	21.1 (21.8)	24.2 (25.1) **
**# of times sex with partner in study** **without** **a condom in the last 3-months (mean, SD) (n = 10,415)**	3.0 (10.3)	1.3 (4.7)	2.1 (7.1) **	2.5 (9.3)	4.5 (13.6) **
**Sex with study partner** **with** **a condom in past 3-months (n = 17,558)**	9227 (87%)	2295 (88%)	1851 (88%)	2195 (87%)	2886 (87%)
**Sex with study partner** **without** **a condom in past 3-months (n = 10,654)**	3198 (30%)	630 (24%)	574 (27%)*	753 (30%)	1241 (37%) **
**Outside sex partner (n = 8790)**	554 (6%)	85 (5%)	61 (4%)	140 (6%)	268 (8%) **

**p = <0.0001

*p<0.05

no asterisks = p>0.05 comparing men who want a child vs. those who do not by serostatus

Women who were HIV+ with a HIV- partner (M-F+) and wanted children were younger (mean age of 27.7 vs. 30.8, p<0.0001), lived with their partner fewer years (5.1 vs. 7.4 years, p<0.0001), had fewer pregnancies at baseline (2.7 vs. 3.7 mean pregnancies, p<0.0001), and fewer children (0.9 vs. 1.7 mean live children, p<0.0001) compared to HIV+ women in M-F+ couples who did not want children. During study follow-up, condomless sex was higher among HIV+ women in M-F+ couples in which the woman wanted children (mean condomless sex acts 4.8 vs. 2.7, p<0.0001) compared to HIV+ women in M-F+ couples who did not want children. The proportion of sex acts without a condom (36% vs. 33%, p = 0.004) were also higher among women who wanted a child compared to HIV+ women who did not want a child. Genital inflammation (12% vs 9%, p = 0.004), outside partners (2% vs. 1%, p = 0.001), and incident pregnancies during the study follow-up (9% vs. 6%, p<0.0001) were more frequent in HIV+ women who wanted children compared to HIV+ women who did not want children. (Table 1)

Among M+F- couples, 45% of HIV+ men wanted a child, compared with 56% of HIV- men in M-F+ couples. HIV+ men who wanted a child tended to be younger (33.0 vs. 38.1, p<0.0001), live with their partner fewer years (5.7 vs. 10.4 years, p<0.0001), had fewer live children (1.4 vs. 2.7 mean children, p<0.0001) and fewer previous pregnancies (2.7 vs. 4.3 mean pregnancies, p<0.0001) compared with HIV+ men who did not want a child. During study follow-up, condomless sex was reported more frequently in follow-up intervals with HIV+ men who wanted a child (27% vs. 24% of intervals included at least one condomless sex act [p = 0.02], and 2.1 vs. 1.3 mean condomless sex acts in the past 3-months, p<0.0001). However, HIV+ men who wanted a child also reported higher mean frequency of condom use in the last 3-months (20.4 vs. 18.8, p = 0.01). HIV+ men who wanted more children had higher rates of genital ulcers (14% vs. 11%, p = 0.04). (Table 2)

HIV- men who wanted a child tended to be younger (32.7 vs. 38.0, p<0.0001), had fewer live children (0.9 vs. 1.8 mean children, p<0.0001), and a partner with fewer pregnancies (2.7 vs. 3.9 mean pregnancies, p<0.0001) compared to HIV- men who did not want a child. HIV- men in M-F+ couples who wanted a child reported higher mean frequency of condomless sex acts in the past 3-months (13.6 vs. 9.3 mean condomless sex acts, p<0.0001) and more frequent sex with condoms in the past 3-months (24.2 vs. 21.1 mean sex acts with condoms, p<0.0001). HIV- men who wanted children had more outside partners (8%) compared to HIV- men who did not want children (6%, p<0.0001). (Table 2)

### Factors associated with wanting a child

In a multivariable model woman’s younger age (aOR per year increase = 0.97, 95% CI = 0.94, 1.00), fewer years cohabiting (aOR = 0.97, 95% CI = 0.94, 1.00), male partner wanting children (aOR = 2.89, 95% CI = 2.14, 3.91), being pregnant at baseline (aOR = 4.80, 95% CI = 2.93, 7.85), and fewer previous pregnancies (aOR = 0.85, 95% CI = 0.78, 0.93) were associated with a woman wanting a child in the future compared to women who did not want a child adjusting for woman’s literacy, years cohabiting, and HIV status at baseline.

Men’s younger age (aOR per year increase = 0.96, 95% CI = 0.93, 0.99), fewer years cohabiting (aOR = 0.95, 95% CI = 0.92, 0.98), and female partner wanting to have children (aOR = 3.00, 95% CI = 2.21, 4.07) were associated with men wanting a child, after adjusting for woman’s age, number of previous pregnancies, baseline pregnancy, HIV status, and men’s literacy. Woman’s HIV status was not associated with wanting to have a child (aOR = 1.2, 95% CI = 0.90, 1.62), however, HIV+ men reported wanting a child less frequently than their HIV negative counterparts (aOR = 0.75, 95% CI = 0.56, 1.02, p = 0.054), after adjusting for age, number of children, and literacy. HIV status was not associated with wanting a child in the women’s model (aOR = 1.20, 95% CI = 0.90, 1.62; p = 0.22) (Table 3).

**Table 3:** Predictors of fertility intentions among men and women in HIV serodiscordant couples in Zambia (2005–2012).

	Crude OR (95% CI)	aOR (95% CI)	P-value
**Model 1: Factors associated with women reporting wanting children in the future (n = 958 couples)** *			
**Woman age (per year increase)**	0.91 (0.90, 0.93)	0.97 (0.94, 1.00)	0.051
**Woman can read Nyanja (vs. illiterate)**	0.73 (0.56, 0.94)	0.89 (0.62, 1.26)	0.508
**Years cohabiting (per year increase)**	0.90 (0.88, 0.92)	0.97 (0.94, 1.00)	0.082
**Man wants (more) children**	2.81 (2.66, 2.97)	2.89 (2.14, 3.91)	< .0001
**Pregnant at baseline (vs. not pregnant)**	3.89 (2.55, 5.94)	4.80 (2.93, 7.85)	< .0001
**HIV status (woman HIV+)**	1.43 (1.14, 1.78)	1.20 (0.90, 1.62)	0.219
**Number of previous pregnancies (per # of pregnancy increase)**	0.72 (0.68, 0.77)	0.85 (0.78, 0.93)	0.001
**Model 2: Factors associated with men reporting wanting children in the future (n = 960 couples)** **			
**Man age (per year increase)**	0.91 (0.90, 0.93)	0.96 (0.93, 0.99)	0.006
**Woman age (per year increase)**	0.90 (0.88, 0.92)	1.00 (0.93, 0.99)	0.9443
**Man can read Nyanja (vs. illiterate)**	0.73 (0.57, 0.94)	0.80 (0.60, 1.07)	0.127
**Years cohabiting (per year increase)**	0.89 (0.87, 0.91)	0.95 (0.92, 0.98)	0.001
**Woman wants (more) children**	2.54 (2.42, 2.67)	3.00 (2.21, 4.07)	< .0001
**Partner pregnant at baseline (vs. not pregnant)**	1.60 (1.09, 2.35)	0.77 (0.50, 1.20)	0.25
**HIV status (man HIV+)**	0.65 (0.51, 0.83)	0.75 (0.56, 1.01)	0.054
**Number of previous pregnancies (per # of pregnancy increase)**	0.72 (0.67, 0.77)	0.90 (0.82, 0.98)	0.017

*198 couples missing one or more indicator

**196 couples missing one or more indicator

### Fertility intentions association with HIV acquisition

In 454 M+F- couples there were 87 female incident infections (incidence rate = 7.43 per 100 couple years, 95% CI = 5.99, 9.12) and in 743 M-F+ couples, there were 95 male incident infections (incidence rate = 5.90, 95% CI = 4.80, 7.18). Fertility intentions were collected at baseline, before HIV acquisition in the model. We found that HIV-negative women in relationships where either or both partners wanted more children were at increased risk of HIV compared to their counterparts who did not want children. Women’s HIV acquisition was associated with the woman wanting a child in the next 12-months (aHR = 2.36, 95% CI = 1.41, 3.96), and beyond 12-months (aHR = 1.99, 95% CI = 1.21, 3.30) adjusted for woman’s age, literacy, and number of previous pregnancies. When the man wanted a child the risk of HIV acquisition among women was more than double that of couples in which the man did not want a child (aHR = 2.34, 95% CI = 1.33, 4.11), and almost double if the man wanted a child in more than 12-months (aHR = 1.83, 95% CI = 1.13, 2.94) adjusted for woman’s age, literacy, and number of previous pregnancies. In a separate model, we found that incident pregnancy was similarly associated with HIV acquisition in M+F- couples (aHR = 1.82, 95% CI = 1.08, 1.88) controlling for woman’s age and years cohabiting (not tabled).

Agreement among partners in the couple about wanting to have a child similarly increased the risk of female HIV acquisition (aHR = 2.08, 95% CI = 1.01, 4.30) when compared with couple agreement that they do not want a child adjusting for woman’s age, literacy, and number of live children. Male seroconversion was also associated with fertility intentions when the woman wanted a child in the next year (aHR = 1.94, 95% CI = 1.02, 3.68) and when both partners wanted a child (aHR = 2.02, 95% CI = 1.09–3.73) adjusted for man’s age, literacy, couple income, number of live children and male circumcision status. (Table 4). Since couples counseling and testing is associated with reduction in HIV incidence [23], survival models included a variable accounting for the first follow-up interval vs. subsequent follow-up intervals.

**Table 4:** Results of multivariable Cox proportional hazard regression of association between fertility intentions on HIV acquisition in: (1) women, (2) men, and (3) in men and women1.

	Adjusted hazard ratio (95% CI)
**1. Woman’s HIV Acquisition**	
**Woman’s fertility intention**	
Woman wants a child in the next 12-months	2.36 (1.41, 3.96)
Woman wants a child but not in the next 12-months	1.99 (1.21, 3.30)
**Man’s fertility intention**	
Man wants a child in the next 12-months	2.34 (1.33, 4.11)
Man wants a child but not in the next 12-months	1.83 (1.13, 2.94)
**Couple fertility intention**	
Couple agrees that they want a child (vs. agrees they do not)	2.08 (1.01, 4.30)
Couple disagrees that they want a child (vs. agrees they do not)	1.41 (0.68, 2.94)
**2. Man’s HIV Acquisition**	
**Woman’s fertility intention on man’s HIV acquisition**	
Woman wants a child in the next 12-months	1.94 (1.02, 3.68)
Woman wants a child but not in the next 12-months	1.59 (0.83, 3.02)
**Man’s fertility intention on man’s HIV acquisition**	
Man wants a child in the next 12-months	1.14 (0.59, 2.21)
Man wants a child but not in the next 12-months	1.71 (0.82, 3.57)
**3. Couple’s fertility intention**	
Couple agrees that they want a child (vs. agrees they do not)	2.02 (1.09, 3.73)
Couple disagrees that they want a child (vs. agrees they do not)	2.00 (0.85, 4.72)

(1) Adjusted for women’s age, women’s literacy, and # of previous pregnancies (n = 545 M+F- couples, 87 female incident infections); (2) Adjusted for men’s age, men’s literacy, couple income, # of live children, and male circumcision status men (n = 575 M-F+ couples, 95 male incident infections); (3) Adjusted for both men and women’s age, men and women’s literacy, and number of live children (n = 1029 couples). All models adjusted for first follow-up interval vs. subsequent follow-up intervals. **Bold: p<0.05**

## Discussion

HIV- women in serodiscordant couples were particularly vulnerable to HIV acquisition when either they or their partner wanted to have a child, especially in the next 12-months. When both partners agreed that they wanted to have a child, the woman and man’s risk of acquiring HIV was also higher than that of couples who agreed they did not want a child. In addition, HIV acquisition was associated with incident pregnancy in our analysis. Our analysis demonstrated that one-third of couples had men and women who did not express the same fertility intentions when interviewed separately. However, disagreement about fertility intentions was not associated with HIV acquisition among women and men. Joint counseling for couples with at least one partner expressing intention for a pregnancy is needed to assist both partners in negotiating a common goal and selecting which safer conception method may work best for them [24,25].

The predictors of wanting to have a child among men and women were similar to previous studies in the region, including younger woman’s age [26], fewer children [25–32], and years married or cohabiting [33]. Similar to a study in Ethiopia, we found that serostatus was associated with decreased fertility intention in men, though this association was attenuated after adjusting for age, literacy, number of live children, partner’s wanting more children, and partner pregnant at baseline [32]. Our study confirmed findings from other studies that each partner’s intention for pregnancy was correlated with the other’s [21, 32–34]. A Kenyan qualitative study of serodiscordant couples found that values and preferences of the couple as a unit may mediate fertility decision making in serodiscordant couples [29]. Similarly, we found that there was a slightly higher risk of HIV acquisition among women and men who agreed with their partner about wanting a child in the future. In our recent publication, factors associated with seroconversion included uncircumcised men, genital inflammation, bilateral inguinal adenopathy and having a STI [21]. Overall HIV incidence in our study was 7.43/100 CY among females and 5.90/100 CY in men, which is lower than a meta-analysis that reported a median annual risk of HIV transmission 11.1 per 100-person years across sub-Saharan Africa [35].

The evidence on the relationship between pregnancy and HIV acquisition are conflicting, especially in the context of widespread ART use in high HIV prevalence settings [21, 35]. A recent study in South Africa showed that pregnancy had a protective effect on HIV acquisition, but young women who had more than 2 pregnancies were at higher risk of acquiring HIV compared to older women [35]. A meta-analysis did not find increased HIV acquisition risk in pregnant or postpartum women relative to non-pregnant/non-lactating women [36]. Our study demonstrated that, in separate models, both fertility intentions and incident pregnancy were associated with increased risk of HIV acquisition in women. We are unable to decipher whether this is due to a biological mechanism associated with pregnancy or because pregnancy is a proxy for condomless sex. More research is needed to better understand the interaction between fertility intentions, behavior and incident pregnancy and the impact on HIV seroconversion.

Our study did not evaluate knowledge of contraception, other family expectations of having a child, nor the influence of health care providers on contraceptive uptake. However, other researchers demonstrated that relatives’ expectations were associated with wanting a child among Ugandan serodiscordant couples [28]. In the same Ugandan study, knowledge of contraception was associated with decreased fertility intention, while knowledge of ART effectiveness was associated with increased fertility intention 28]. In a study of HIV+ women in South Africa, only 41% of women had communicated with providers about future pregnancy options [30]. Further, high rates of unintended pregnancy have been reported among HIV+ women [37, 38]. Those studies, along with our findings, highlight the urgent need for integrating family planning, and interventions for safer conception into HIV care and treatment [29, 38]. Another study evaluated a Safer Conception Toolkit for HIV care and treatment providers and HIV discordant couples trying to conceive in Kenya [39]. A recent study in South Africa found high uptake of safer contraception methods suggesting that combination prevention methods are acceptable to clients and appropriate for scale-up [40].

### Limitations

Because these couples self-selected into the cohort after couples’ counseling and testing, they have inherent differences from the general population. Participants in this study were cohabiting, mutually-disclosed serodiscordant couples. As a result, their risk behavior was likely very different from that of couples who have not tested together and mutually disclosed. Participants were also screened and treated for STIs which may have further reduced transmission risk. Another important limitation is that fertility intention was only reported at baseline, and fertility intention may change over time. Stigma around having children among HIV+ women may have led to under-reporting fertility intentions. Social desirability bias could have resulted from familiarity between nurse counselor and study participant during the interview. As a result of these limitations, information bias due to exposure variables captured as self-reported is possible. Selection bias may also have affected the study as couples who stayed in the cohort may differ in terms of their health-seeking behavior, or other demographic or behavioral data, from similar couples who were not enrolled or were lost to follow up, or separated. Further there were biases in retention in care, as living further from the clinic, younger age, and women’s age at first intercourse were predictive of attrition [11]. High loss to follow-up may have led to an under-estimation of the true incidence in the population if couples who seroconverted did not come back for study visits, though our exposure, fertility intentions, were associated with retention in care.

## Conclusions

Our analysis demonstrated that women were particularly vulnerable to HIV acquisition when either they and/or their partner wanted to have a child in the next 12-months. Men were also at risk of HIV acquisition when their partner wanted a child or if they both agreed that they wanted a child. Optimizing delivery of HIV risk reduction strategies during peri-conception periods (e.g. safer conception) requires understanding how HIV serodiscordant couples and HIV affected and infected individuals approach fertility decisions. Our study highlights the importance of pregnancy planning and safer conception services as a critical, but oftentimes neglected, component of HIV care and treatment.
